# Signalment and Blood Types in Cats Being Evaluated as Blood Donors at Two Italian University Blood Banks

**DOI:** 10.1155/2014/704836

**Published:** 2014-03-16

**Authors:** Eva Spada, Arianna Miglio, Daniela Proverbio, Maria Teresa Antognoni, Giada Bagnagatti De Giorgi, Elisabetta Ferro, Vittorio Mangili

**Affiliations:** ^1^Veterinary Transfusion Unit (REV), Department of Health, Animal Science and Food Safety (VESPA), University of Milan, Via G. Celoria 10, 20133 Milan, Italy; ^2^Veterinary Transfusion Unit (EMOVET-UNIPG), Department of Veterinary Clinical Science, University of Perugia, Via San Costanzo 4, 06124 Perugia, Italy

## Abstract

Data from potential feline blood donors presented at two university blood banks in Italy were recorded. Blood typing was performed using an immunochromatographic method. Over the three years of the study 357 cats representing 15 breeds, 45.3% female and 54.7% male, with a mean age of 3.8 years were evaluated. Of these 90.5% were blood type A, 5.6% type B, and 3.9% type AB. The majority of the cats (54.6%) were European DSH (92.3% were type A, 5.1% type B, and 2.6% type AB), and 21% were Maine Coon (MCO) cats (100% blood type A). The estimated frequencies of transfusion reactions following an unmatched transfusion between DSH (donors and recipients), MCO (donor and recipients), DSH donors and MCO recipients, and MCO donors and DSH recipients were 4.8%, 0%, 0%, and 5.1% for major reactions and 7.2%, 0%, 7.7%, and 0% for minor transfusions reactions, respectively. In a population of blood donors that includes DSH and MCO the risk of transfusion reaction is between 5% and 8% if typing is not performed on donor and recipient blood. Blood typing should therefore be performed before transfusion to remove the risk of transfusion reactions due to blood type incompatibilities.

## 1. Introduction 

The AB blood system is the major feline blood system and consists of the 3 blood types A, B, and AB [1]. All cats older than three months have naturally occurring alloantibodies against the other blood types, with the exception of type AB cats [2]. For this reason (and because there is no universal feline donor), all cats must be blood-typed and receive transfusions of the same blood type to prevent major and minor transfusion reactions. Transfusion of compatible blood prevents hemolytic transfusion reactions, including fatal major reactions when cats with type B blood receive type A or AB blood transfusions. In addition, the vitality and life span of transfused RBCs can be seriously affected by minor transfusion reactions when cats with type A blood receive type B or AB blood transfusions, and cats with type AB blood receive type B blood transfusions [2, 3]. As transfusion reactions are not only dependent on the AB system blood type compatibility but can also occur due to other blood types (e.g., Mik) [4] and to WBCs, platelets, or plasma protein [5], a cross-matching should always be performed before a transfusion.

Feline transfusion medicine has advanced in recent decades resulting in an increasing number of cats receiving blood transfusions and being evaluated as potential blood donors [6–9]. Transfusions can be life-saving in critically ill veterinary patients. It is valuable to have data on the frequencies of the different blood types in cats from different geographic areas to decide which feline breeds should be screened and included in blood donor programmes. Feline breeds in which are present subjects of all three AB blood types are useful to be chosen as blood donors to have the availability of blood or blood components of all AB system blood types.

This retrospective study reports the signalment and distribution of blood type AB system in cats being evaluated as potential blood donors at two Italian university blood banks and assesses the risk of major and minor transfusion reactions due to AB blood type system incompatibility in this population.

## 2. Materials and Methods

Data (sex, breed, and age) from potential feline blood donors presented at the University blood banks in Milan (northern Italy) and in Perugia (central Italy) between September 2010 and June 2013 were recorded. Jugular or cephalic venipuncture was performed and approximately 2 mL of blood collected into EDTA tubes and stored at 4–6°C. Blood was drawn from all cats without sedation. Blood typing was performed within 48 hours of collection using an immunochromatographic cartridge method (LabTEST A + B, Alvedia, Lyon, France). Consent for blood sampling and analysis was given by the cat owners.

The chance of a major transfusion reaction was calculated by multiplying the percentage of type A and AB donor cats by the percentage of type B recipient cats. The chance of a minor transfusion reaction was calculated by multiplying the percentage of type B and AB donor cats by the percentage of type A recipient cats and adding the percentage of type B donor cats multiplied by the percentage of type AB recipient cats [7].

## 3. Results 

There were 357 cats included in the study, 187 (52.4%) from the blood bank in Milan and 170 (47.6%) from Perugia, representing 15 different breeds: 158 were female (45.3%) and 191 were male (54.7%) (in eight cats sex was not recorded), with a mean age of 3.8 years (SD±2.9, range 2–8 years). Of these 90.5% (*n* = 323) were blood type A, 5.6% (*n* = 20) type B and 3.9% (*n* = 14) type AB. Numbers of each breed evaluated and their blood types are reported in Table 1.

The majority of the cats (*n* = 195; 54.6%) were European DSH, of which 92.3% (*n* = 180) were blood type A, 5.1% (*n* = 10) blood type B, and 2.6% (*n* = 5) blood type AB. Maine Coon (MCO) cats made up 21% of the total (*n* = 75), and 100% of these were blood type A; 7% of the cats (*n* = 25) were Ragdolls, of which 68% (*n* = 17) were type A, 8% (*n* = 2) type B, and 24% (*n* = 6) type AB.

The estimated frequencies of transfusion reactions following an unmatched transfusion between DSH (donors and recipients), MCO (donor and recipients), DSH donors and MCO recipients, and MCO donors and DSH recipients were 4.8%, 0%, 0%, and 5.1% for major reactions and 7.2%, 0%, 7.7%, and 0% for minor transfusions reactions, respectively.

## 4. Discussion 

This study aimed to report the signalment and distribution of blood types A, B and AB in cats being evaluated as potential blood donors and to assess the risk of major and minor transfusion reactions due to AB blood type system incompatibility; the frequencies of the three feline blood types in cats evaluated in this study are comparable to those previously reported worldwide, with a predominance of blood type A [9, 10].

The commercial immunochromatographic cartridge blood typing test used in this study has previously been evaluated and shown to be an accurate (94.8%) typing method, with a high sensitivity and specificity in feline AB system blood typing [11]. In this study we have not reported on Mik red cell antigen blood type as there is no commercial test to test this feline blood type [4].

An important observation from this study is that this is the first time a significant number of MCO cats (*n* = 75) were blood-typed and all were blood type A. If this finding is confirmed in a larger number of MCO cats, then this breed presents no risk of transfusion reaction related to the feline AB blood system if MCO cats are selected as both donors and recipients. In contrast, when a DSH is used at random as a donor for a MCO cat, the risk of transfusion reaction for the recipient could reach 7.7%. In addition if a MCO cat is used as a blood donor for a DSH without blood typing, the risk of a major transfusion reaction is 5.1%. This data reaffirms that blood typing is mandatory before transfusion in order to remove the risk of transfusion reactions due to AB blood system type incompatibilities. Even if MCO in our survey had only blood type A, this result would never justify not blood typing MCO cats if used as a donor or if they are in need of a blood transfusion.

Although limited numbers of Ragdolls (*n* = 25) and Persians (*n* = 8) were evaluated in our study these groups showed the highest prevalence of AB blood type (24% and 37.5%, resp.). As plasma from type AB blood cats lacks both anti-A and anti-B alloantibodies [2, 5], plasma from cats with type AB blood could be used in emergency situations when type A or B plasma is not available, making these cats ideal plasma donors.

The results of blood typing in this study show MCO cats to be ideal blood donors in the general feline population in which blood type A is the prevalent blood type, whilst Ragdoll and Persian cats make ideal donors for plasma products as these cats have a high prevalence of AB blood type [7]. When MCO, Ragdolls, and Persian cats are considered as blood donors their genetic predisposition for hypertrophic cardiomyopathy (HCM) must be considered [12]. Although the collection of a unit of blood for transfusion from healthy cats weighing more than 5 kg appears to be safe, it does lead to a decrease in arterial blood pressure, PCV, and heart rate [13], that could pose a risk to cats with cardiac diseases. However, any cat can be affected by HCM and cats with mild HCM may be asymptomatic [14]. For this reason all potential donor cats should be screened with echocardiography and have their blood pressure evaluated before entering a blood donor programme and at least on an annual basis. Because hypertrophic cardiomyopathies are difficult to diagnose and may occult, and such cats can experience acute death posttransfusion, in donor cats cardiomyopathy has been ruled out by cardiac echo before each blood donation.

## Figures and Tables

**Table 1:** Breed and blood type in 357 cats evaluated as potential blood donors at two Italian university blood banks.

Breed	*n* (%)	Type A *n* (%)	Type B *n* (%)	Type AB *n* (%)
European domestic shorthair	195 (54.6)	180 (92.3)	10 (5.1)	5 (2.6)
Maine Coon	75 (21)	75 (100)	0 (0)	0 (0)
Ragdoll	25 (7)	17 (68)	2 (8)	6 (24)
Persian	8 (2.2)	4 (50)	1 (12.5)	3 (37.5)
Sphynx	7 (2.0)	5 (71.4)	2 (28.6)	0 (0)
Norwegian forest cat	7 (2.0)	7 (100)	0 (0)	0 (0)
Birman	7 (2.0)	7 (100)	0 (0)	0 (0)
Devon Rex	6 (1.7)	3 (50)	3 (50)	0 (0)
British shorthair	6 (1.7)	6 (100)	0 (0)	0 (0)
Abyssinian	6 (1.7)	4 (66.7)	2 (33.3)	0 (0)
Chartreux	5 (1.4)	5 (100)	0 (0)	0 (0)
Siberian	3 (0.8)	3 (100)	0 (0)	0 (0)
Siamese	3 (0.8)	3 (100)	0 (0)	0 (0)
Exotic shorthair	3 (0.8)	3 (100)	0 (0)	0 (0)
Russian blue	1 (0.3)	1 (100)	0 (0)	0 (0)
